# Supplementary material for: Design and validation of HIV peptide pools for detection of HIV-specific CD4+ and CD8+ T cells
Source: PLoS One. 2022 Aug 16;17(8):e0268370. doi: 10.1371/journal.pone.0268370 (PMC9380920; doi:10.1371/journal.pone.0268370)
Supplement: S1 Table — (PDF) [file pone.0268370.s001.pdf]

Supplemental Table 1. List of HIV class I peptides.

sequence	length	protein	Mapped Start Position	Mapped End Position
GELDRWEKI	9	Gag	11	19
KIRLRPGGKK	10	Gag	18	27
IRLRPGGKKKY	11	Gag	19	29
RLRPGGKKKYKL	12	Gag	20	31
RPGGKKKYKLK	11	Gag	22	32
KYKLKHIVW	9	Gag	28	36
HLVWASRELERF	12	Gag	33	44
ELRSLYNTV	9	Gag	74	82
RSLYNTVATLY	11	Gag	76	86
TLYCVHQK	8	Gag	84	91
IEIKDTKEAL	10	Gag	92	101
NSSKVSQNY	9	Gag	124	132
VQNLQGQMV	9	Gag	135	143
HQAISPRTLNAW	12	Gag	144	155
VKVIEEKAF	9	Gag	156	164
EEKAFSPEVI	10	Gag	160	169
KAFSPEVIPMF	11	Gag	162	172
EVIPMFSAL	9	Gag	167	175
SEGATPQDL	9	Gag	176	184
TPQDLNMML	9	Gag	180	188
GHQAAMQML	9	Gag	193	201
KETINEEAAEW	11	Gag	202	212
AEWDRVHPV	9	Gag	210	218
HPVHAGPIA	9	Gag	216	224
GQMREPRGSDI	11	Gag	226	236
TSTLQEQIGW	10	Gag	240	249
NPPIPVGDIY	10	Gag	253	262
EIYKRWII	8	Gag	260	267
KRWIILGLNK	10	Gag	263	272
RRWIQLGLQK	10	Gag	263	272
GLNKIVRMY	9	Gag	269	277
VRMYSPVSI	9	Gag	274	282
YSPVSILDI	9	Gag	277	285
FRDYVDRFFKTL	12	Gag	293	304
DRFYKTLRA	9	Gag	298	306
AEQASQEVKNWM	12	Gag	306	317
VKNWMTETL	9	Gag	313	321
DCKTILKAL	9	Gag	329	337
ACQGVGGPGHK	11	Gag	349	359
GPGHKARVL	9	Gag	355	363
AEAMSQVTNS	10	Gag	364	373
CRAPRKKGC	9	Gag	405	413

TERQANFL	8	Gag	427	434
RQANFLGKI	9	Gag	429	437
FLGKIWPSYK	10	Gag	433	442
KELYPLTSL	9	Gag	481	489
RVKEKYQHL	9	gp160	2	10
AENLWVTVYY	10	gp160	31	40
TVYYGVPVWK	10	gp160	37	46
VPVWKEATTTL	11	gp160	42	52
LFCASDAKAY	10	gp160	52	61
KAYETEVHNVW	11	gp160	59	69
DPNPQEVVL	9	gp160	78	86
MHEDIISLW	9	gp160	104	112
SVITQACPK	9	gp160	199	207
SFEPIPIHY	9	gp160	209	217
CAPAGFAIL	9	gp160	218	226
RPNNNTRKSI	10	gp160	298	307
HIGPGRAFY	9	gp160	310	318
RGPGRAFVTI	10	gp160	311	320
EIIGDIRQAY	10	gp160	321	330
SFNCGGEFF	9	gp160	375	383
LPCRIKQII	9	gp160	416	424
RIKQIINMW	9	gp160	419	427
RAIEAQQHM	9	gp160	557	565
QTRVLAIERYL	11	gp160	577	587
ERYLKDQQLL	10	gp160	584	593
TAVPWNASW	9	gp160	606	614
VFAVLSIVNR	10	gp160	698	707
IVNRNRQGY	9	gp160	704	712
RLRDLLLIVTR	11	gp160	770	780
IVTRIVELL	9	gp160	777	785
GRRGWEALKY	10	gp160	786	795
KYCWNLLQY	9	gp160	794	802
QELKNSAVSL	10	gp160	805	814
SLLNATDIAV	10	gp160	813	822
EVAQRAYR	8	gp160	831	838
IPRRIRQGL	9	gp160	843	851
RIRQGLERA	9	gp160	846	854
RQGLERALL	9	gp160	848	856
WPTVRERM	8	Nef	13	20
RMRRAEPAA	9	Nef	19	27
LEKHGAITS	9	Nef	37	45
FPVTPQVPLR	10	Nef	68	77
RPQVPLRPMTY	11	Nef	71	81
RPQVPLRPMTYK	12	Nef	71	82
PLRPMTYKAA	10	Nef	75	84
LRPMTYKAAL	10	Nef	76	85

KAAVDLSHFL	10	Nef	82	91
KAAVDLSHFLK	11	Nef	82	92
GAFDLSFFL	9	Nef	83	91
FLKEKGGL	8	Nef	90	97
KEKGGLEGL	9	Nef	92	100
KRQDILDLWVY	11	Nef	104	115
RRQDILDLWVY	11	Nef	105	115
HTQGYFPDWQNY	12	Nef	116	127
TQGYFPDWQNYT	12	Nef	117	128
YFPDWQNYTP	10	Nef	120	129
YTPGPGIRY	9	Nef	127	135
TPGPGVRYPL	10	Nef	128	137
TRYPLTFGW	9	Nef	133	141
RYPLTFGWCY	10	Nef	134	143
YPLTFGWCYKL	11	Nef	135	145
VLEWRFDSRL	10	Nef	180	189
WRFDSRLAF	9	Nef	183	191
NSPTRREL	8	Pol	24	31
ITLWQRPLV	9	Pol	59	67
DTVLEEWNL	9	Pol	86	94
EEMNLPGRW	9	Pol	90	98
RQYDQILIEI	10	Pol	113	122
GKKAIGTVLV	10	Pol	124	133
LVGPTPVNI	9	Pol	132	140
TPVNIIGRNML	11	Pol	136	146
FPISPIETV	9	Pol	155	163
IETVPVKL	8	Pol	160	167
GPKVKQWPL	9	Pol	173	181
ALVEICTEMEK	11	Pol	188	198
KLVDFRELNK	10	Pol	228	237
GIPHPAGLK	9	Pol	248	256
TVLDVGDAY	9	Pol	262	270
VPLDEDFRKY	10	Pol	273	282
YTAFTIPSV	9	Pol	282	290
NETPGIRYQY	10	Pol	292	301
IRYQYNVL	8	Pol	297	304
LPQGWKGSPA	10	Pol	304	313
SPAIFQSSM	9	Pol	311	319
AIFQSSMTK	9	Pol	313	321
KQNPDIVIY	9	Pol	328	336
HPDIVIYQY	9	Pol	330	338
NPEIVIYQY	9	Pol	330	338
VIYQYMDDL	9	Pol	334	342
IEELRQHLL	9	Pol	357	365
IVLPEKDSW	9	Pol	399	407
LVGKLNWASQIY	12	Pol	415	426

QIYPGIKVR	9	Pol	424	432
YPGIKVRQL	9	Pol	426	434
IPLTEEAEL	9	Pol	448	456
ILKEPVHGVY	10	Pol	464	473
GQGQWTYQI	9	Pol	488	496
IYQEPFKNLK	10	Pol	496	505
RMRGAHTNDVK	11	Pol	511	521
IAMESIVIW	9	Pol	530	538
PIQKETWETW	10	Pol	547	556
GAETFYVDGAANR	13	Pol	591	603
ETKLGKAGY	9	Pol	604	612
IVTDSQYALGI	11	Pol	650	660
QIIEQLIKK	9	Pol	675	683
LFLDGIDKA	9	Pol	715	723
LPPIVAKEI	9	Pol	743	751
THLEGKIIL	9	Pol	781	789
HVASGYIEA	9	Pol	793	801
IEAEVIPAET	10	Pol	799	808
HTDNGSNF	8	Pol	829	836
STTVKAACWW	10	Pol	838	847
IQQEFGIPY	9	Pol	850	858
VRDQAEHL	8	Pol	880	887
KTAVQMAVF	9	Pol	888	896
AVFIHNFKRK	10	Pol	894	903
FKRKGGIGGY	10	Pol	900	909
GERIVDII	8	Pol	912	919
IIATDIQTK	9	Pol	918	926
KIQNFRVYY	9	Pol	934	942
VPRRKAKII	9	Pol	975	983
RKAKIIRDY	9	Pol	978	986
KAVRLIKFLY	10	Rev	14	23
QAVRIIKILY	10	Rev	14	23
ERILSTYLGR	10	Rev	57	66
RPAEPVPLQL	10	Rev	66	75
CCFHCQVC	8	Tat	30	37
FQTKGLGISYGR	12	Tat	38	49
RIRTWKSLVK	10	Vif	17	26
HMYISKKAK	9	Vif	28	36
ISKKAKGWF	9	Vif	31	39
HPRVSSEVHI	10	Vif	48	57
IPLGDAKLII	10	Vif	57	66
WHLGHGVSI	9	Vif	79	87
LGHGVSIEW	9	Vif	81	89
LADQLIHLHY	10	Vif	102	111
KTKPPLPSVKK	11	Vif	158	168
EAVRHFPRIW	10	Vpr	29	38

AVRHFPRIWL	10	Vpr	30	39
FPRIWLHGL	9	Vpr	34	42
ETYGDTWTGV	10	Vpr	48	57
DTWAGVEAIIR	11	Vpr	52	62
AIIRILQQL	9	Vpr	59	67
YRLGVGALI	9	Vpu	5	13
EYRKILRQR	9	Vpu	29	37
