# Supplementary material for: Design and validation of HIV peptide pools for detection of HIV-specific CD4+ and CD8+ T cells
Source: PLoS One. 2022 Aug 16;17(8):e0268370. doi: 10.1371/journal.pone.0268370 (PMC9380920; doi:10.1371/journal.pone.0268370)
Supplement: S2 Table — (PDF) [file pone.0268370.s002.pdf]

Supplemental Table 2. List of HIV class II peptides.

sequence	length	protein	Mapped Start Position	Mapped End Position
NCSFNATTVVRDRDQ	15	env	156	170
NATTVVRDRKQTVYA	15	env	160	174
VYALFYRLDIVPLTK	15	env	172	186
RIHIGPGRAFYTTKN	15	env	308	322
TGDIIGDIRQAHCNI	15	env	319	333
GIIGDIRQAHCHVSR	15	env	321	335
GAINTTNETFRPGGG	15	env	459	473
RQANFLGKIWPSNKG	15	gag	429	443
GRPGNFLQSRPEPTA	15	gag	443	457
ESFRSGVETTTPPQK	15	gag	461	475
EPIDKELYPLASLRS	15	gag	477	491
DKELYPLASLRSLFG	15	gag	480	494
TEKLWVTVYYGVPVW	15	gp160	31	45
WVTVYYGVPVWKDAE	15	gp160	35	49
GATTTLFCASDAKAY	15	gp160	47	61
TTLFCASKAKAYDTE	15	gp160	50	64
THACVPADPNPQEMV	15	gp160	71	85
ENVTENFNMWKNEMV	15	gp160	87	101
ENFNMWKNEMVNQMQ	15	gp160	91	105
EMVNQMQEDVISLWD	15	gp160	99	113
SLKPCVKLTPLCVSL	15	gp160	115	129
CVKLTPLCVTLECRN	15	gp160	119	133
KNCSFNISTSIRGKV	15	gp160	155	169
DKMQKEYALLYKLDI	15	gp160	167	181
INCNTSAITQACPKV	15	gp160	194	208
VITQACPKVSFEPIP	15	gp160	200	214
PKVTFDPIPIHYCTP	15	gp160	206	220
FDPIPIHYCTPAGYA	15	gp160	210	224
PAGFAILKCNNKTFN	15	gp160	220	234
TNVSTVQCTHGRPIY	15	gp160	240	254
RPVVSTQLLLNGSLA	15	gp160	252	266
PVVSTQLLLNGSLAE	15	gp160	253	267
EVVIRSANFTDNAKT	15	gp160	269	283
IRSENITNNAKTIIV	15	gp160	272	286
SANFTDNAKTIIVQL	15	gp160	274	288
RIQRGPGRAFVTIGK	15	gp160	308	322
EQRGPGRAFVTIGKI	15	gp160	309	323
GRAFVTIGKIGNMRQ	15	gp160	314	328

CNISRAQWNNTLEQI	15	gp160	331	345
SSGGKPEIVTHSFNC	15	gp160	364	378
PEIVTHSFNCGGEFF	15	gp160	369	383
GEFFYCNSTQLFNST	15	gp160	380	394
EFFYCNTTQLFNNTW	15	gp160	381	395
TQLFNSTWFNSTWST	15	gp160	388	402
TWFNSTWSTKGSNNT	15	gp160	394	408
TWSTKGSNNTEGSDT	15	gp160	399	413
SSSIITIPCRIKQII	15	gp160	410	424
TIPCRIKQIINMWQE	15	gp160	415	429
IIKQIINMWQEVGRA	15	gp160	419	433
RIKQIINMWQKVGKA	15	gp160	419	433
KQIINMWQKVGQAMY	15	gp160	421	435
INMWQEVGKAMYAPP	15	gp160	424	438
VGRAMYAPPIKGNIT	15	gp160	430	444
PIKGNITCKSNITGL	15	gp160	438	452
GNSNNESEIFRPGGG	15	gp160	459	473
ETFRPGGGDMRNNWR	15	gp160	466	480
DMRDNWRSELYKYKV	15	gp160	474	488
ELYKYKVVEIKPLGV	15	gp160	482	496
YKVVEIKPLGVAPTT	15	gp160	486	500
GIVQQQNNLLRAIEA	15	gp160	547	561
QQHLLQLTVWGIKQL	15	gp160	562	576
LTVWGIKQLQARVLA	15	gp160	568	582
GIKQLQARVLAVERY	15	gp160	572	586
EQIWNHTTWMEWDRE	15	gp160	620	634
EESQNQQEKNEQELL	15	gp160	647	661
ASLWNWFNITNWLWY	15	gp160	667	681
IKLFIMIVGGLVGLR	15	gp160	682	696
RTFIMIVGGLIGLRI	15	gp160	683	697
FVVLSIVNRVRQGYS	15	gp160	699	713
DRVIEVVGQAYRAIR	15	gp160	827	841
DRVIEVVQGAYRAIR	15	gp160	827	841
AIRHIPRRIRQGLER	15	gp160	839	853
HIPRRIRQGLERILL	15	gp160	842	856
GKWSKSSIVGWSAVR	15	nef	3	17
WSKSSIVGWPAVRER	15	nef	5	19
LEKHGAITSSNTAAT	15	nef	37	51
SSNTAATNAACAWLE	15	nef	45	59
EVGFPVIPQVPLRPM	15	nef	65	79
FPVRPQVPLRPMTYR	15	nef	68	82
QVPLRPMTYKGALDL	15	nef	73	87
AVDLSHFLKEKGGLM	15	nef	84	98

GWFLKEKGGLDGLIY	15	nef	88	102
SWFLKEKGGLEGLIY	15	nef	88	102
GWPFKLVPVDPREVQ	15	nef	140	154
EVLMWKFDSRLAFHH	15	nef	179	193
VLEWRFDSRLAFHHV	15	nef	180	194
RWEFDSSLARRHLAR	15	nef	182	196
LAFHHVARELHPEYF	15	nef	189	203
IAPGQMREPRGSDIA	15	pol	223	237
MREPRGSDIAGTTST	15	pol	228	242
GSDIAGTTSTLQEQI	15	pol	233	247
STLQEQIGWMTNNPP	15	pol	241	255
EQIAWMTSNPPVPVG	15	pol	245	259
NPPIPVGEIYKRWII	15	pol	253	267
PVGEIYKRWIILGLN	15	pol	257	271
YKRWIILGLNKIVRM	15	pol	262	276
ILGLNKIVRMYSPVS	15	pol	267	281
IVRMYSPVSILDIKQ	15	pol	273	287
SPTSILDIRQGPKEP	15	pol	278	292
IRQGPKEPFRDYVDR	15	pol	285	299
GPKEPFRDYVDRFYK	15	pol	288	302
FRDYVDRFYKTLRAE	15	pol	293	307
DRFYKTLRAEQASQE	15	pol	298	312
TLRAEQASQEVKNWM	15	pol	303	317
RAEQASQEVKNWMTE	15	pol	305	319
LLVQNANPDCKTILR	15	pol	321	335
ILKALGPAATLEEMM	15	pol	333	347
LGPAATLEEMMTACQ	15	pol	337	351
PSHKARVLAEAMSQA	15	pol	356	370
QRGNFRNQRKTVKCF	15	pol	379	393
FNCGKEGHTARNCRA	15	pol	393	407
KEGHQMKDCTERQAN	15	pol	418	432
MKDCTERQANFLGKI	15	pol	423	437
QRPLVTIKIGGQLKE	15	pol	495	509
TPVNIIGRNLLTQIG	15	pol	568	582
NFPISPIETVPVKLR	15	pol	586	600
TEMEKEGKISKIGPE	15	pol	626	640
PSGLKKKKSVTVLDM	15	pol	684	698
FRKYTAFTIPSINNE	15	pol	711	725
GSPAIFQSSMTKILE	15	pol	742	756
SPAIFQSSMTKILEP	15	pol	743	757
IGQHRTKIEELRQHL	15	pol	782	796
KDSWTVNDIQKLVGK	15	pol	836	850
SWTVNDIQKLVGKLV	15	pol	838	852

VHDIQKLVGKLNWAS	15	pol	841	855
QKLWGKLNWASQIYP	15	pol	845	859
WRQLCKLLRGTKALT	15	pol	863	877
GTKALTEVIPLTEEA	15	pol	872	886
PLTEEAELELAENRE	15	pol	881	895
LAENREILKEPVHGV	15	pol	890	904
TYQIYQEPFKNLKTG	15	pol	925	939
QEPFKNLKTGKYAKM	15	pol	930	944
YAKMRSAHTNDVKQL	15	pol	941	955
GKTPKFKLPIQKETW	15	pol	971	985
IPEWEFVNTPPLVKL	15	pol	998	1012
WEFVNTPPLVKLWYQ	15	pol	1001	1015
LEKEPIVGAETFYVD	15	pol	1016	1030
KEPIVGAETFYVDGA	15	pol	1018	1032
EKVYLAWVPAHKGIG	15	pol	1116	1130
KVYLAWVPAHKGIGG	15	pol	1117	1131
HSNWRAMASDFNLPP	15	pol	1163	1177
GKIILVAVHVASGYI	15	pol	1217	1231
VTSGYIEAEVIPAET	15	pol	1226	1240
HLKTAVQMAVYIHNF	15	pol	1318	1332
KTAVQMAVFIHNFKR	15	pol	1320	1334
AGERIVDIIATDIQT	15	pol	1343	1357
QKQITKIQNFRVYYR	15	pol	1361	1375
ISKFRVYYRDSRDPI	15	pol	1367	1381
SDIKVVPRRKAKIIR	15	pol	1402	1416
DEELIRTVRLIKLLY	15	rev	9	23
SNPPPNPEGTRQARR	15	rev	25	39
SYHCLVCFQTKGLGI	15	tat	31	45
ITKALGISYGRKKRR	15	tat	39	53
KALGISYGRKKRRQR	15	tat	41	55
DHQNLISKQPLPRTQ	15	tat	64	78
SLQYLALVALVAPKK	15	vif	144	158
RHFPRIWLHGLGQHI	15	vpr	32	46
PRIWLHGLGQHIYET	15	vpr	35	49
ETYGDTWAGVEAIIR	15	vpr	48	62
DTWAGVEAIIRILQQ	15	vpr	52	66
EAIIRILQQLLFIHF	15	vpr	58	72
QLLFIHFRIGCRHSR	15	vpr	66	80
IHFRIGCRHSRIGVT	15	vpr	70	84
VLAIVALVVATIIAI	15	vpu	5	19
